# Atlanta Academy of Medicine

**Published:** 1871-09

**Authors:** Edwin P. Ingraham


### ATLANTA ACADEMY OF MEDICINE.

Reported by Edwin P. Ingraham, M.D.

July 28th, 1871.

Dr. Logan, President, in the chair.

Dr. J. G. Westmoreland called the attention of the Academy to the use of carbolic acid in the treatment of menorrhagia. He had recently treated two cases, in which the hemorrhage, having continued for a week, was promptly relieved by one or two doses. He gave two grains of the acid, in half a tumbler of water, every three or four hours. His attention was first directed to the use of carbolic acid, as a hæmostatic, by Dr. Harden's recommending it in purpura hemorrhagica. Dr. Westmoreland thought his success met with in these two cases, though not sufficient to substantiate the entire effectiveness of carbolic acid as a hæmostatic, marked it as worthy of further trial.

Dr. Cumming.—Was the hemorrhage attended by ovarian irritation?

Dr. Westmoreland replied that he was not positive on that point—the hemorrhage recurring at intervals of a week or so, and continuing from one to two weeks.

Dr. Harden stated that he had used carbolic acid in hæmaturia and hemorrhage of the lungs—in two-grain doses, freely diluted—and found it a very prompt hæmostatic for any internal hemorrhage.

Dr. Bozeman remarked, that it was evident that the mass of women do not mature but one ovum per month, and that he was led to think that the ovaries acted alternately from finding that the tenderness of the ovaries alternated monthly. Some women menstruate every two weeks, withont any disturbance of the general health. He would like to know, in such cases, if this semi-monthly menstruation might not be a normal process, and not a diseased condition.

Dr. Wells observed that menorrhagia occurred in both the plethoric and anæmic, and that the condition of the system should govern our treatment. In cases attended with anæmia, he was accustomed to stimulate. Port wine and alum, given freely, he thought most efficacious; in addition, he sometimes found it necessary to use quinine. In the plethoric, he usually found cupping over the spine to afford the speediest relief. He had used carbolic acid in diarrhea and tuberculosis, as a disinfectant, but not in larger doses than half a grain every two or three hours, and not a hæmostatic.

Dr. W. F. Westmoreland reported a case, now under his care, for the purpose of eliciting an expression of opinion in regard to it from the Academy. A lady, aged twenty-four, rather frail and slender, had not menstruated for four years: for three years, there was no show at all; once during the last year, and once this, she has had a mucous discharge slightly tinged with blood. Until within a year, she suffered no inconvenience, being in apparently good health, and not until the last two months has she suffered excessively, during which time she has had hysterical convulsions and frequent nervous attacks, tonic contractions of the muscles, etc. On examination, July 27th, he found the womb to be half its normal size, high up, and retroverted. On attempting to introduce a small probe, he found it impossible to pass the internal os. The patient attributes the primary cause of the trouble to exposure to cold during a menstrual period, causing a sudden cessation, four years ago; previous to that time, she had menstruated for six years regularly. Dr. Westmoreland inquires, "Are the ovaries at fault?" He thinks not, as she has pain over the ovaries, and other symptoms that usually precede a regular period, every twenty-eight days. These symptoms, of late, occur more frequently. "What is the matter, and what the treatment?"

Dr. Cumming inquired if the patient was well-developed?

Dr. Westmoreland replied she was, and in a perfect state of health up to the cessation of the menses.

Dr. Harden asked if there had been any vicarious menstruation, or symptoms of consumption?

Dr. Westmoreland.—Two or three times, has had hemorrhage from the nose; no symptoms of consumption. Her sisters are healthy and stout.

Dr. Cumming remarked, that very young women do often cease menstruation without any premonitory symptoms, except debility. This frequently occurs with school girls striving for superior mental attainments, and ambitious of obtaining high standing in their class. He mentioned, as a case in point, a young girl whom he knew, whose health had been ruined at school. She had not menstruated for three years, and had not been in good health since. Also, he knew of several cases where menstruation had ceased on entering school, and resumed during vacation.

Dr. Westmoreland stated that, in the case he had just reported, there was no history of hereditary disease. It was a sudden cessation during good health. He is of the opinion the trouble must have been local.

Dr. J. G. Westmoreland remarked, that it seemed to him to be a plain case of inactivity of the womb—inertia; it may have been engorgement or atrophy, causing loss of function.

Dr. W. F. Westmoreland observed that, after pregnancy, the uterus gradually became reduced in size. If this diminution or absorption ceases too early, we have *sub*involution; if this process continues too long, we have *super*involution, or atrophied womb, and there is a loss of function. He was of the opinion that the case reported is of a similar character; the uterus has become atrophied, from some cause not known, while the ovaries continue to act.

Dr. Bozeman, having been called upon, said it was very difficult to form an opinion without a personal examination of the patient. It seems there has been a suppression for about four years,—from what cause, it is difficult to ascertain; it may have been from nervous excitement, or retroversion may have occurred at the time, and there may have been some cervical inflammation resulting in occlusion. The facts are, at present, that we have to deal with an atrophied organ; these organic difficulties have to be overcome, in addition, of course, to attention to general health and hygene.

Dr. W. F. Westmoreland stated that, in consultation with Dr. Logan, it had been agreed to dilate the os with tents, and then to introduce a small sound every few days, to arouse the organ; also, to try electricity by use of the electric pessary.

Dr. Bozeman suggested that the wearing of the horse-shoe pessary would aid in stimulating the uterus.

Dr. Cumming remarked that Dr. Bozeman had spoken more especially of local treatment. He would expect to find poor blood. Menstruation was an extra performance, and it required but a slight cause to stop it. Whenever there was a want of good blood, menstruation failed. There are a vast number of women in whom the menstrual function is more or less deranged. A woman ought to feel just as well during the menstrual period as at other times. As soon as the blood becomes damaged, menstruation is diminished, and it suggests that the blood be looked after, in addition to local treatment.

Dr. Bozeman inquired what was the vocation of the patient?

Dr. Westmoreland.—She is a woman in moderate circumstances, living in the country, and attends to some domestic duties; not accustomed to much mental exercise.

